# What makes for a vibrant medical physics professional society?

**DOI:** 10.1002/acm2.12207

**Published:** 2017-10-29

**Authors:** Per Halvorsen

**Affiliations:** ^1^ Lahey Hospital & Medical Center Burlington MA USA

This issue's invited Editorial is provided by Per Halvorsen, Associate Editor‐in‐Chief of the JACMP. It concerns the recent effort to modify the Association's governance and contains some very perceptive analysis that should prove useful if the AAPM decides to revisit this issue at some future date — Michael Mills, JACMP EIC

Those of us who are AAPM members have just witnessed a robust debate about the normally dry topic of Association governance. A proposal for a significant change in the Association's structure was developed by leaders of the Association with the help of a paid consulting firm, and put to the membership for a vote on By‐Laws changes needed to implement the proposed governance change. The discussion leading up to the vote was energetic, with many thoughtful observations. The proposal failed to gain the support of the necessary 2/3rds majority of voting members, despite being the result of a sincere effort by a group of dedicated volunteer members.

So, what are the key ingredients for a vibrant medical physics professional society? I posit that three ingredients are crucial: diversity of perspective, grass roots engagement, and fiscal checks and balances.

One of the threads in the AAPM governance debate could be described as “experience vs. diversity of perspective”. Experience certainly brings value — and in the context of a professional society, this is typically measured in terms of volunteer service within the organization. A disproportionate focus on volunteer experience as a criterion for Association leadership positions, however, brings an increased likelihood of entrenched priorities leading to a less flexible organization. Without diversity of perspective, the organization risks continuing practices even when they do not best serve the organization or its members. One of the comments in the AAPM discussion used the phrase “constructive turbulence”, which captures the principle quite nicely. For example, the AAPM continued to contract with AIP Publishing respecting journal and society publications for over 4 decades; after opening up for competitive bidding, we now have a contract with one of the leaders in scientific publishing with the potential of realizing significantly more net revenue flowing to the Association. The American College of Medical Physics (ACMP) was formed in part due to frustration over entrenched priorities within the AAPM and the consequent lack of focus on professional‐practice issues. The ACMP's formation motivated the AAPM to become more engaged in professional‐practice issues, leading to a more active and productive Professional Council. This Journal was founded during the ACMP's tenure — its open access model challenged the traditional approach to medical physics publishing and has contributed to a more diverse perspective on the Journals’ business management now that the ACMP's functions have been assumed by the AAPM.

Diversity of perspective, then, is arguably more important than volunteer experience for Board of Directors positions in a medical physics professional society — and I say that as one of the individuals holding the distinction of many years of AAPM volunteer experience. Another comment in the recent AAPM governance debate was: “Governance needs to systematically and structurally enforce the possibility that different points of view can and will be presented.” I agree with this statement. What can be done structurally to ensure that different points of view are presented? This leads to the second crucial ingredient: grass roots engagement.

The AAPM's existing governance structure strongly supports grass roots engagement. The Chapter Representatives to the Board of Directors are selected by their local Chapter members — perhaps the essence of grass roots engagement. And while the Association has a Nominating Committee which makes a good‐faith effort to identify strong candidates for leadership positions, it is impossible for a small group of individuals to know all the members of the Association. The result is that the Nominating Committee, however well intentioned (full disclosure: I'm a current member of the Committee), is likely to miss many potentially excellent candidates for such leadership positions. In this context, I believe it is essential to preserve the ability for direct nomination by the membership for certain Board positions — which our current governance allows. In the last round of elections, the Nominating Committee's slate of candidates for At‐Large Board positions consisted entirely of PhD physicists. This was not an intentional bias by the Committee, but we missed the mark in terms of ensuring a sufficiently diverse slate of candidates. The membership corrected this by nominating several, equally well qualified, candidates. Our governance structure ensured grass roots engagement, and for that I am grateful.

Finally, fiscal checks and balances. An important principle in this regard, as described in another comment in the AAPM governance debate, is that the organization's budget should be approved by individuals who do not have a direct role in the use of the allocated funds. Our current governance structure honors this principle. The majority of allocated funds are used by the Councils and their many Committees and Task Groups, and by the AAPM Headquarters operation. The representatives of both entities (Council Chairs and Executive Director) are nonvoting members of the Board in the current structure — essentially, advisors who can inform the Board's deliberations but cannot vote.

Some have advocated for aligning the AAPM's governance more closely with the structure of our physician sister societies, such as the ACR and ASTRO. I believe this is misplaced. We have a strong working relationship with them, as we should, but we are different — we do not have the operating budgets of our physician sister societies. Rather than aspiring to become more similar to such societies, we should embrace what has made our Association strong and nurture the key ingredients for an even more vibrant society: diversity of perspective, grass roots engagement, and fiscal checks and balances. If a new AAPM governance change is proposed in the future, it is my hope that it will be crafted with robust grass roots involvement and structured with these key ingredients firmly in mind.

## CONFLICT OF INTEREST

The author declares no conflict of interest.

